# Transcatheter Versus Surgical Aortic Valve Replacement in Women of Childbearing Age in the United States

**DOI:** 10.1016/j.shj.2025.100746

**Published:** 2025-10-24

**Authors:** Mahmoud Ismayl, Hasaan Ahmed, Andrew M. Goldsweig, Mayra Guerrero

**Affiliations:** a Department of Cardiovascular Medicine, Mayo Clinic, Rochester, Minnesota, USA; b Department of Cardiovascular Medicine, Baystate Medical Center and Division of Cardiovascular Medicine, University of Massachusetts-Baystate, Springfield, Massachusetts, USA

**Keywords:** Aortic stenosis, Outcomes, Readmissions, SAVR, TAVR, Women

## Abstract

**Background:**

Women of childbearing age occasionally require aortic valve replacement (AVR), sometimes performed with transcatheter AVR (TAVR). Outcomes of TAVR versus surgical AVR (SAVR) in women of childbearing age have not been evaluated. We aimed to evaluate the contemporary use and outcomes of TAVR versus SAVR in women of childbearing age in the United States.

**Methods:**

Women aged 18-50 years hospitalized for isolated AVR were identified in the Nationwide Readmissions Database (2016-2022). In-hospital outcomes of TAVR versus SAVR were compared using propensity-score matching. Readmissions were compared using the Cox proportional hazards regression model.

**Results:**

Of 6926 weighted hospitalizations for isolated AVR in women aged 18-50 years, 897 (13.0%) included TAVR, and 6029 (87.0%) included SAVR. From 2016-2022, the proportion of AVR performed using TAVR increased from 7.4% to 16.3% in women aged 18-50 years (p_trend_<0.001). Compared with SAVR, TAVR was associated with lower in-hospital mortality (<1.4 vs. 3.5%, *p* = 0.03), acute kidney injury (9.0 vs. 16.8%, *p* = 0.002), and need for blood transfusion (7.1 vs. 19.1%, *p* ​< ​0.001), but higher heart block (23.5 vs. 9.7%, *p* ​< ​0.001) and vascular complications (5.0 vs. 2.1%, *p* = 0.03). Length of stay was shorter (2 vs. 7 days, *p* ​< ​0.001) and nonhome discharges were lower (16.2 vs. 56.7%, *p* ​< ​0.001) with TAVR compared with SAVR. Ninety-day all-cause readmissions were similar between TAVR and SAVR (12.6 vs. 13.3%, *p* = 0.78).

**Conclusions:**

This nationwide observational analysis found that TAVR is increasingly performed among women aged 18-50 years with lower in-hospital mortality and resource utilization and similar readmissions compared with SAVR.

## Highlights

• Data are limited on the optimal aortic valve replacement strategy for women of childbearing age (18-50 years).
• Transcatheter aortic valve replacement (TAVR) is increasingly performed in women aged 18-50 years and is offered to patients with more comorbidities than those undergoing surgical aortic valve replacement (SAVR).
• Among women aged 18-50 years, TAVR was associated with lower in-hospital mortality and resource utilization and similar 90-day readmissions compared with SAVR.
• Randomized controlled trials exploring the long-term safety and effectiveness of TAVR versus SAVR among women of childbearing age are warranted.

## Graphical abstract

TAVR vs. SAVR in propensity score-matched women aged 18-50 years in the United States. A) Year-over-year trend of TAVR vs. SAVR in women aged 18-50 years; B) comparison of 90-day all-cause readmissions following TAVR vs. SAVR in women aged 18-50 years; C) comparison of TAVR vs. SAVR in-hospital outcomes in women aged 18-50 years. ∗*p* ​< ​0.05. ∗∗*p* ​≥ ​0.05. ^†^Propensity score-matched based on age, primary payer, median income quartile, hospital location (urban/rural) and teaching status, number of hospital beds, admission type (elective/nonelective) and day (weekend/weekday), bicuspid aortic valve, Elixhauser and Charlson Comorbidity Index scores, and relevant comorbidities (Supplemental Table 4). aHR = adjusted hazard ratio; AVR ​= ​aortic valve replacement; NRD = Nationwide Readmissions Database; PPM = permanent pacemaker; SAVR = surgical aortic valve replacement; TAVR = transcatheter aortic valve replacement.

**Figure:**
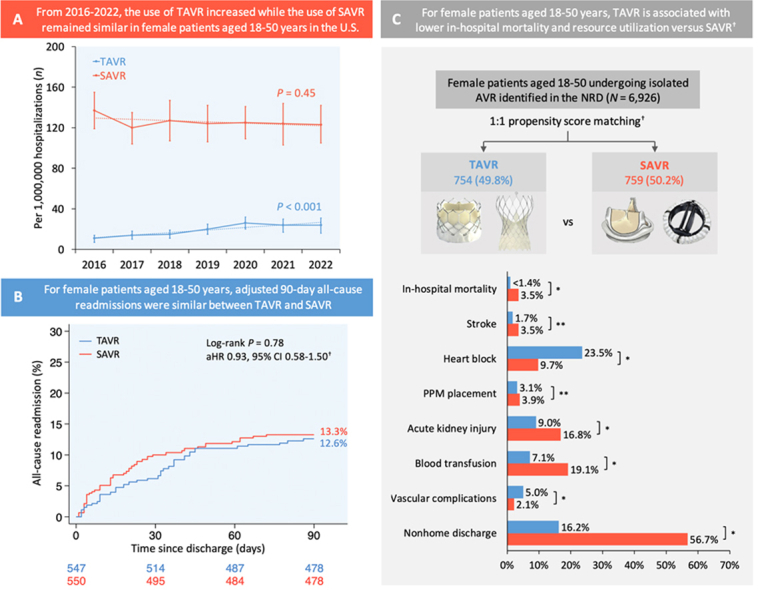

## Introduction

Severe aortic stenosis (AS) is one of the most prevalent valvular heart lesions, often necessitating intervention via transcatheter aortic valve replacement (AVR) (TAVR) or surgical AVR (SAVR).^1^, ^2^, ^3^ Although SAVR had historically been the only treatment for AS, the use of TAVR has expanded significantly following its Food and Drug Administration approval in 2011.^2^^,^^4^ Landmark randomized controlled trials (RCTs) have established the safety and efficacy of TAVR across high-, intermediate-, and low-risk patient populations, leading to its expanded use beyond initial indications.^2^^,^^5^, ^6^, ^7^ Nevertheless, current guidelines continue to recommend SAVR for patients younger than 65 years, primarily due to concerns about the long-term durability of transcatheter bioprosthetic valves and the complexities of future valve reintervention following TAVR.^8^^,^^9^ Despite these recommendations, national trends reveal a growing preference for TAVR among younger adults, coinciding with a steady decline in SAVR volumes.^7^

Women of childbearing age constitute a particularly understudied and vulnerable subgroup within the broader younger adult population.^10^ Women of childbearing age who receive mechanical heart valves require lifelong anticoagulation with warfarin. Warfarin is associated with teratogenic effects, particularly during the first trimester of pregnancy, and switching to alternative anticoagulation strategies such as low molecular weight heparin or unfractionated heparin increases the risk of prosthetic valve thrombosis. Consequently, pregnancy in women with mechanical valves is considered high risk and requires multidisciplinary management and careful planning.^11^^,^^12^ Limited data exist regarding the most suitable valve replacement modality for women of childbearing age, particularly in light of reproductive considerations, anticipated patient longevity, and unknown differences in long-term outcomes between TAVR and SAVR.^13^^,^^14^ Notably, the RHEIA (Randomized researcH in womEn all comers wIth Aortic stenosis) trial demonstrated that in women with severe AS, TAVR was associated with a lower incidence of the composite outcome of death, stroke, or rehospitalization at 1 year compared with surgery, although the mean age of participants was 73 years.^15^ Similarly, the VIVA (transcatheter aortic valve replacement versus surgical aortic valve replacement for treating elderly patients with severe aortic stenosis and small aortic annuli) trial, which predominantly enrolled women (93%) with severe AS and small aortic annulus, found no significant differences in outcomes between TAVR and SAVR after a median follow-up of 2 years; however, the mean participant age was 75.5 years.^16^ Therefore, we queried the Nationwide Readmissions Database (NRD) to evaluate the use and short-term outcomes of TAVR versus SAVR in women of childbearing age in the United States.

## Methods

### Data Source and Ethics Statement

Hospitalization data were abstracted from the NRD, which is part of the Healthcare Cost and Utilization Project (HCUP) family of databases sponsored by the Agency for Healthcare Research and Quality.^17^ The NRD is the largest publicly available, fully deidentified, all-payer inpatient health care readmission database in the United States. The NRD is compiled from billing data submitted by hospitals to statewide organizations across the United States and has reliable and verified patient linkage numbers that can be used to track patients across hospitals within each state and calendar year while adhering to strict privacy guidelines. The NRD covers approximately 18 million unweighted hospitalizations each year with a diverse patient population, growing from 27 states in 2016 to 30 states in 2022 (Supplemental Table 1).^17^ When weighted, the NRD extrapolates to the national level, representing approximately 35 million hospitalizations each year. The unweighted sample represents approximately 50% of all US hospitalizations. The NRD contains both patient- and hospital-level information. Up to 40 discharge diagnoses and 25 procedure codes are collected for each patient using *International Classification of Diseases, Tenth Revision* (*ICD-10*) codes.^18^ The NRD captures all admissions and readmissions with nationally representative weighting, allowing the analysis of readmission rates. Each patient is assigned a unique identifier code using the variable “NRD_VistLink” to track patients within a calendar year. The NRD days-to-event variable (“NRD_DaysToEvent”) is used to capture readmissions within a calendar year but not across different years.^17^ The design and methodology of the NRD have been described previously.^19^^,^^20^, ^21^, ^22^ This study followed the STROBE (Strengthening the Reporting of Observational Studies in Epidemiology) reporting guideline (Supplemental Table 2)^23^ and was exempt from the requirements of the Mayo Clinic Institutional Review Board because the NRD is a fully deidentified, Health Insurance Portability and Accountability Act-compliant database that is publicly available from the HCUP website (www.hcup-us.ahrq.gov).

### Study Population and Patient Selection

**Figure 1:**
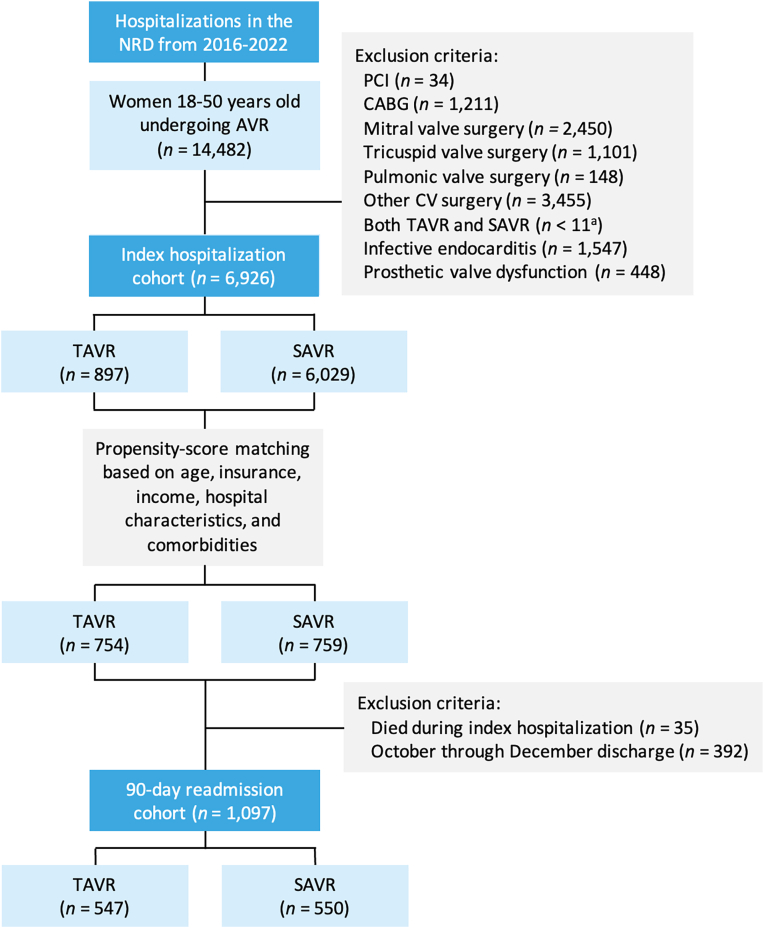
**Study flow diagram showing inclusion and exclusion criteria.** Hospitalization counts represent national-level estimates. Abbreviations: AVR, aortic valve replacement; CABG, coronary artery bypass grafting; CV, cardiovascular; NRD, Nationwide Readmissions Database; PCI, percutaneous coronary intervention; SAVR, surgical aortic valve replacement; TAVR, transcatheter aortic valve replacement.

We searched the NRD from January 2016 through December 2022 to identify hospitalizations in which women aged 18-50 years underwent isolated AVR with TAVR (*ICD-10*, Procedure Coding System 02RF37H, 02RF38H, 02RF3JH, 02RF3KH, X2RF332, 02RF37Z, 02RF38Z, 02RF3JZ, and 02RF3KZ in any procedural field) or SAVR (*ICD-10*, Procedure Coding System 02RF07Z, 02RF08Z, 02RF0JZ, 02RF0KZ, and X2RF032 in any procedural field). We excluded hospitalizations in which the patient was aged <18 years or >50 years as well as those with concomitant percutaneous coronary intervention, concomitant surgeries (coronary artery bypass grafting, mitral/tricuspid/pulmonic valve surgeries, aortic surgeries, other cardiovascular surgeries), both TAVR and SAVR during the same admission, infective endocarditis, or prosthetic valve dysfunction (Figure 1). A complete list of *ICD-10* diagnosis and procedure codes used in this study is presented in Supplemental Table 3.

When evaluating 90-day readmissions, we excluded hospitalizations in which the patient died during the index hospitalization as well as any discharge that occurred after September 30 of each calendar year because the NRD follows patients within a single calendar year and does not capture readmissions across calendar years (Figure 1). Therefore, to enable a full 90-day follow-up for all discharges, we only used data from January 1 to September 30 of each year for the analysis on 90-day readmissions. In patients who had multiple 90-day readmissions, only the first readmission was included in the analysis. The NRD does not provide data on out-of-hospital deaths, and therefore patients who died at home within 90 days would be counted as patients without a readmission within 90 days.

### Study Outcomes

Temporal trends in isolated TAVR and SAVR use in women aged 18-50 years were reported. The primary outcome was in-hospital all-cause mortality. Secondary outcomes included in-hospital complications of stroke, heart block, permanent pacemaker (PPM) implantation, acute kidney injury (AKI), major bleeding, need for blood transfusion, and vascular complications (defined as a composite of arteriovenous fistula, aneurysm, hematoma, retroperitoneal bleeding, and venous thromboembolism), as well as hospital length of stay (LOS), total hospital costs (inflation adjusted to 2022 US dollars),^24^ and discharge disposition. Charge-to-cost ratio files were used to convert charges to costs at the individual hospital level. All-cause and heart failure readmissions at 90 days were also evaluated.

### Statistical Analysis

Normality of continuous data was assessed using the Shapiro–Wilk or Kolmogorov–Smirnov test, as appropriate. Continuous variables were reported as mean ​± ​SD for normal distributions or median and interquartile range for skewed distributions. Categorical variables were reported as percentages. Comparisons were made between the 2 groups using the Student’s *t* test or Mann–Whitney *U* test for continuous variables and the Pearson χ2 or Fisher exact test for categorical variables, as appropriate.

Temporal trends in hospitalizations for TAVR and SAVR were analyzed using linear regression. Propensity-score matching methodology was performed to match women aged 18-50 years who underwent isolated TAVR to those who underwent isolated SAVR, respectively, in a 1:1 ratio. Each case was propensity score matched to a control using nearest neighbor technique with a caliper width of 0.2 (Supplemental Figures 1-4). The propensity score was calculated from the following variables: age, primary payer, median income quartile by ZIP code, hospital location (urban/rural) and teaching status, number of hospital beds, admission type (elective/nonelective) and day (weekend/weekday), bicuspid aortic valve, Elixhauser and Charlson Comorbidity Index scores, and relevant comorbidities (Supplemental Table 4) using R’s MatchIt package.^25^ Adjustment variables were selected *a priori* on the basis of their clinical significance and on their likely influence on in-hospital outcomes and readmissions. Logistic regression analysis was performed to estimate odds ratios with 95% CIs.

The probability of 90-day readmission, stratified on the basis of TAVR versus SAVR, was graphically displayed using the Kaplan-Meier method and was compared using the log-rank test. Cox proportional hazards regression analysis was performed to estimate hazard ratios with their corresponding 95% CIs. The assumptions of Cox proportional hazards regression were graphically assessed using log-log plots and tested based on Schoenfeld residuals.

Complete data were available for all variables except for primary payer (missing 0.1%), median household income quartile by ZIP code (missing 1.0%), and type of admission (missing 0.1%). As the overall missing values were minimal (<1.5%) and limited to only 3 of the potential confounding variables, they were assumed to be missing at random, and the level of bias was likely small. Missing values were handled with listwise deletion and were not included in the regression analysis.

For all statistical analyses, a 2-tailed *p* ​< ​0.05 was considered statistically significant. Given the large sample size, not all statistically significant *p* values represent clinically significant differences and therefore require careful interpretation. All statistical analyses were performed using Stata version 17 (StataCorp, College Station, TX) software and R software for Statistical Computing, version 4.3 (R Foundation for Statistical Computing, Vienna, Austria), accounting for the NRD sampling design, and were weighted using sampling weights provided with the NRD to estimate national-level effects per HCUP-NRD recommendations similar to prior studies.^17^^,^^26^, ^27^, ^28^, ^29^

## Results

### Patient and Hospital Characteristics

**Figure 2:**
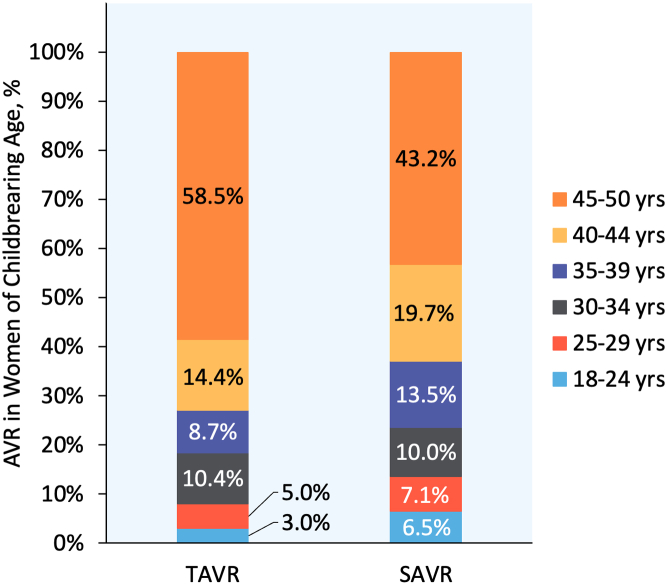
**Age distribution of women of childbearing age undergoing AVR.** Abbreviations: AVR, aortic valve replacement; SAVR, surgical aortic valve replacement; TAVR, transcatheter aortic valve replacement.

From January 2016 through December 2022, 6926 hospitalizations in the NRD met the inclusion criteria; of which, 897 (13.0%) included TAVR and 6029 (87.0%) included SAVR (Figure 1). Most women of childbearing age undergoing AVR were ≥40 years old (Figure 2). Patients selected for TAVR were older, more likely to have Medicare insurance, and less likely to have a bicuspid aortic valve compared to patients selected for SAVR. They also had higher Elixhauser and Charlson Comorbidity Index scores, which were driven by higher rates of diabetes, hypertension, dyslipidemia, coronary artery disease, congestive heart failure, renal failure requiring dialysis, liver disease, chronic pulmonary disease, obstructive sleep apnea, and cancer. Furthermore, patients selected for TAVR were more likely to have a history of myocardial infarction, stroke/transient ischemic attack, cardiac arrest, percutaneous coronary intervention, coronary artery bypass grafting, or pre-existing implantable cardioverter-defibrillator or PPM. Baseline characteristics of the unmatched and matched cohorts, stratified by TAVR versus SAVR procedures, are shown in Table 1.

**Table 1:** Baseline characteristics stratified by TAVR vs. SAVR

	Unmatched			Propensity-matched∗		
	TAVR (*n* = 897)	SAVR (*n* = 6029)	*p*	TAVR (*n* = 754)	SAVR (*n* = 759)	*p*
Demographic characteristics						
Age (years)	46 (39-49)	43 (35-48)	<0.001	45 (37-49)	45 (38-48)	0.46
18-24	3.0	6.5	<0.001	3.5	2.7	0.29
25-29	5.0	7.1		5.2	6.0	
30-34	10.4	10.0		12.3	8.3	
35-39	8.7	13.5		10.2	11.2	
40-44	14.4	19.7		15.3	21.3	
45-50	58.5	43.2		53.5	50.5	
Primary payer						
Medicare	27.1	10.1	<0.001	21.5	22.9	0.92
Medicaid	24.1	26.1		25.7	24.9	
Private insurance	44.2	57.3		48.1	47.3	
Self-pay	1.5	3.9		1.8	<1.4†	
Other	3.1	2.6		2.9	3.6	
Income quartile‡						
I	27.0	30.6	0.04	28.4	32.1	0.68
II	25.6	27.6		23.7	20.9	
III	29.9	23.6		31.2	31.0	
IV	17.5	18.2		16.7	16.0	
Hospital characteristics						
Location/teaching status						
Rural	7.2	9.5	0.26	8.1	7.6	0.72
Urban nonteaching	91.6	89.0		90.6	90.3	
Urban teaching	1.2	1.5		<1.4†	2.1	
Bed size§						
Small	2.7	6.0	<0.001	2.9	2.1	0.73
Medium	13.2	18.2		14.6	15.2	
Large	84.1	75.8		82.5	82.7	
Elective admission	64.5	72.4	<0.001	66.3	67.0	0.83
Weekend admission	9.8	5.3	<0.001	8.0	9.3	0.51
Clinical characteristics						
Bicuspid aortic valve	13.0	32.6	<0.001	15.3	15.2	0.99
Elixhauser comorbidity index	5 (4-7)	4 (3-6)	<0.001	5 (3-7)	5 (4-7)	0.39
Charlson comorbidity index	2 (1-4)	1 (0-2)	<0.001	2 (1-3)	2 (1-3)	0.87
0	10.9	30.1	<0.001	12.8	9.2	0.34
1	23.4	34.1		26.7	29.0	
2	22.1	18.6		23.8	27.2	
≥3	43.6	17.2		36.7	34.6	
Diabetes mellitus	28.2	15.4	<0.001	25.1	25.4	0.93
Hypertension	62.6	52.6	<0.001	59.4	59.2	0.96
Dyslipidemia	31.9	25.2	0.003	28.4	35.2	0.06
Nicotine/tobacco use	33.1	39.0	0.02	33.5	38.4	0.18
Alcohol abuse	1.6	1.7	0.79	1.4	2.3	0.36
Drug abuse	5.3	8.8	0.02	6.4	6.2	0.94
Obesity	32.6	34.0	0.59	34.4	34.4	0.99
Coronary artery disease	31.7	14.0	<0.001	22.9	23.9	0.75
Peripheral vascular disease	18.4	16.6	0.35	17.7	18.9	0.68
Atrial fibrillation/flutter	12.0	13.7	0.36	10.8	13.2	0.34
Congestive heart failure	73.0	35.7	<0.001	68.7	71.4	0.46
Renal failure	26.8	9.5	<0.001	19.2	19.7	0.87
Dialysis dependent	13.1	2.5	<0.001	6.8	7.8	0.60
Liver disease	9.5	6.0	0.009	8.9	9.7	0.71
Chronic pulmonary disease	27.8	19.4	<0.001	25.3	26.9	0.63
Obstructive sleep apnea	12.0	8.4	0.01	11.4	13.8	0.34
Coagulopathy	15.0	29.7	<0.001	17.5	20.1	0.39
Cancer	3.0	0.8	<0.001	2.2	2.3	0.92
Malnutrition	2.7	2.3	0.57	2.0	2.4	0.69
Depression	16.8	17.7	0.65	17.3	17.4	0.99
Previous history						
Myocardial infarction	5.7	2.4	<0.001	3.3	3.6	0.79
Stroke/TIA	11.3	4.3	<0.001	8.8	10.3	0.48
Cardiac arrest	1.4	0.4	0.01	<1.4†	<1.4†	0.82
PCI	7.5	1.0	<0.001	2.9	2.9	0.99
CABG	7.5	1.2	<0.001	3.0	4.9	0.23
ICD	2.8	0.5	<0.001	2.5	1.9	0.56
PPM	3.4	1.4	0.004	2.6	1.9	0.53

Data presented as median (IQR) or %. Two authors (M.I. and H.A.) independently verified the *International Classification of Diseases, Tenth Revision* ​(*ICD-10*) codes that corresponded to each comorbidity (Supplemental Table 3), and any disagreements in inclusion or exclusion of *ICD-10* codes were discussed with a third author (M.G).

Abbreviations: CABG, coronary artery bypass grafting; HCUP, Healthcare Cost and Utilization Project; ICD, implantable cardioverter-defibrillator; IQR, interquartile range; NRD, Nationwide Readmission Database; PCI, percutaneous coronary intervention; PPM, permanent pacemaker; SAVR, surgical aortic valve replacement; TAVR, transcatheter aortic valve replacement; TIA, transient ischemic attack.

∗ Propensity-matched based on age, primary payer, median income quartile, hospital location (urban/rural) and teaching status, number of hospital beds, admission type (elective/nonelective) and day (weekend/weekday), bicuspid aortic valve, Elixhauser and Charlson Comorbidity Index scores, and relevant comorbidities (Supplemental Table 4).

† Cell counts <11 are not reportable per HCUP guidelines.

‡ Estimated median household incomes are ZIP code–specific, updated annually, and classified into 4 quartiles indicating the poorest to wealthiest populations.

§ Bed-size categories are based on inpatient beds and are specific to the hospital’s location and teaching status. A more detailed explanation of all the variables in the NRD, including the specific dollar amounts in each category of median household income and the number of hospital beds in each category, is available online (https://hcup-us.ahrq.gov/db/nation/nrd/nrddde.jsp).

### Trend Analysis

**Figure 3:**
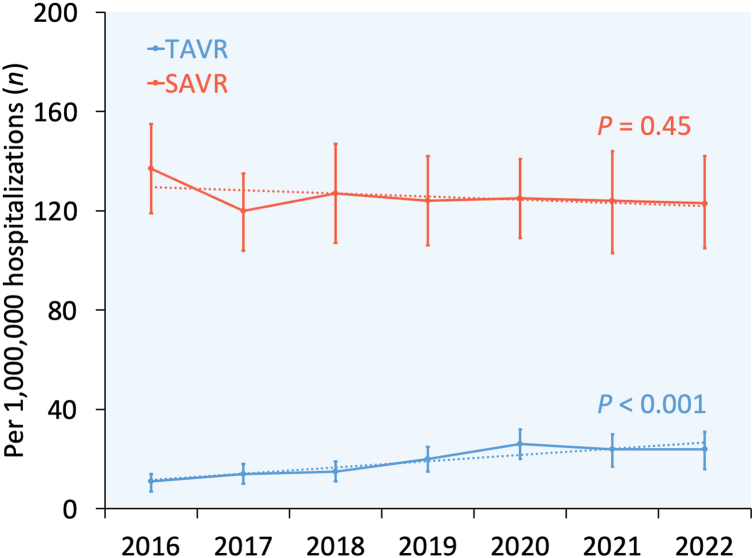
**Year-over-year trend in the use of TAVR vs. SAVR among women aged 18-50 years in the United States from 2016 through 2022.** Error bars represent 95% confidence intervals. Dotted lines represent linear trends. Abbreviations: SAVR, surgical aortic valve replacement; TAVR, transcatheter aortic valve replacement.

From 2016 through 2022, the use of TAVR increased from 11 to 24 per 1,000,000 hospitalizations (p_trend_<0.001), whereas the use of SAVR remained similar (p_trend_ = 0.45) in women aged 18-50 years. By 2022, the proportion of AVR performed using TAVR in women aged 18-50 years reached 16.3%. Annual trends for TAVR versus SAVR in women aged 18-50 years are shown in Figure 3.

### In-Hospital Outcomes

The estimated overall in-hospital all-cause mortality rate was 2.3% (95% CI: 1.4%-3.7%). After propensity score matching 1513 patients (754 TAVR and 759 SAVR), those selected for TAVR had lower rates of in-hospital mortality (<1.4 vs. 3.5%, *p* = 0.03), AKI, and blood transfusion, and higher rates of heart block and vascular complications compared to those selected for SAVR (Table 2).

**Table 2:** In-hospital outcomes stratified by TAVR vs. SAVR

	Unmatched				Propensity-matched∗			
	TAVR (*n* = 897)	SAVR (*n* = 6029)	Odds ratio (95% CI)	*p*	TAVR (*n* = 754)	SAVR (*n* = 759)	Odds ratio (95% CI)	*p*
Mortality	<1.2†	1.7	0.55 (0.20-1.53)	0.25	<1.4†	3.5	0.31 (0.10-0.96)	0.03
Stroke	2.0	3.5	0.56 (0.29-1.06)	0.07	1.7	3.5	0.48 (0.20-1.17)	0.10
Heart block	24.2	12.1	2.31 (1.81-2.96)	<0.001	23.5	9.7	2.87 (1.90-4.33)	<0.001
PPM implantation	3.9	4.7	0.83 (0.50-1.39)	0.48	3.1	3.9	0.80 (0.38-1.67)	0.55
Acute kidney injury	9.8	11.7	0.83 (0.58-1.17)	0.29	9.0	16.8	0.49 (0.31-0.76)	0.002
Major bleeding	1.5	1.1	1.40 (0.61-3.21)	0.43	1.8	1.4	1.32 (0.35-4.95)	0.67
Blood transfusion	6.9	18.2	0.33 (0.23-0.48)	<0.001	7.1	19.1	0.32 (0.20-0.52)	<0.001
Vascular complications	4.9	2.0	2.57 (1.56-4.25)	<0.001	5.0	2.1	2.38 (1.07-5.30)	0.03

Data presented as %.

The *International Classification of Diseases, Tenth Revision* (*ICD-10*) codes corresponding to each of the in-hospital outcomes were identified with the same process used to identify comorbidity codes (Supplemental Table 3).

Abbreviations: HCUP, Healthcare Cost and Utilization Project; PPM, permanent pacemaker; SAVR, surgical aortic valve replacement; TAVR, transcatheter aortic valve replacement.

∗ Propensity-matched based on age, primary payer, median income quartile, hospital location (urban/rural) and teaching status, number of hospital beds, admission type (elective/nonelective) and day (weekend/weekday), bicuspid aortic valve, Elixhauser and Charlson comorbidity index scores, and relevant comorbidities (Supplemental Table 4).

† Cell counts <11 are not reportable per HCUP guidelines.

Patients selected for TAVR had a shorter median hospital LOS (2 vs. 7 days, *p* ​< ​0.001) and similar median total costs ($52,499 vs. $57,691, *p* = 0.60) compared to those selected for SAVR (Table 3). For hospitalizations in which the patient was discharged alive, those selected for TAVR were discharged at greater rates to home without services (83.8 vs. 43.3%, *p* ​< ​0.001) as opposed to home health care, skilled nursing, or intermediate care facilities compared to those selected for SAVR (Table 3).

**Table 3:** Discharge disposition and resource utilization stratified by TAVR vs. SAVR

	Unmatched			Propensity-matched∗		
	TAVR (*n* = 897)	SAVR (*n* = 6029)	*p*	TAVR (*n* = 754)	SAVR (*n* = 759)	*p*
Discharge disposition						
Routine	83.2	51.8	<0.001	83.8	43.3	<0.001
Transfer to short-term hospital	<1.2†	0.6		<1.4†	1.9	
Transfer to SNF or ICF	2.8	5.3		2.7	7.8	
Home health care	13.2	42.3		13.0	47.0	
Resource utilization						
LOS (d)	3 (1-9)	6 (5-10)	<0.001	2 (1-8)	7 (5-14)	<0.001
Hospital cost ($)	64,420 (38,817- 112,413)	70,446 (43,722- 121,000)	0.17	52,499 (37,236-94,698)	57,691 (37,834-101,406)	0.60

Data presented as % or median (IQR).

Abbreviations: HCUP, Healthcare Cost and Utilization Project; ICF, intermediate care facility; IQR, interquartile range; LOS, length of stay; SAVR, surgical aortic valve replacement; SNF, skilled nursing facility; TAVR, transcatheter aortic valve replacement.

∗ Propensity-matched based on age, primary payer, median income quartile, hospital location (urban/rural) and teaching status, number of hospital beds, admission type (elective/nonelective) and day (weekend/weekday), bicuspid aortic valve, Elixhauser and Charlson comorbidity index scores, and relevant comorbidities (Supplemental Table 4).

† Cell counts <11 are not reportable per HCUP guidelines.

### 90-Day Readmission

The estimated overall 90-day all-cause readmission rate was 12.9% (95% CI 12.1-13.8%). The most common cause for 90-day readmission was heart failure (2.1% [95% CI 1.1-3.1%]).

**Figure 4:**
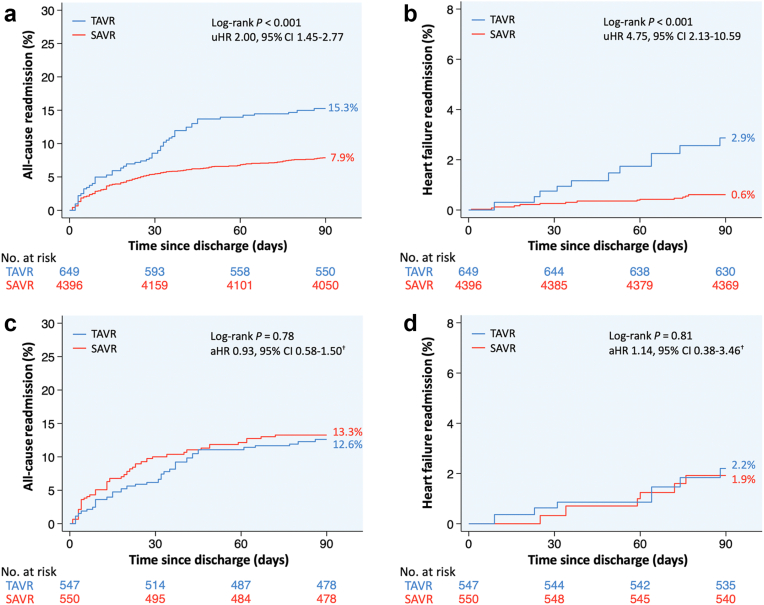
**Kaplan–Meier curves of 90-day readmissions following TAVR vs. SAVR in unmatched (a: all-cause readmissions, b: heart failure readmissions) and propensity score-matched^†^ (c: all-cause readmissions, d: heart failure readmissions) women aged 18-50 years.**^†^Propensity score-matched based on age, primary payer, median income quartile, hospital location (urban/rural) and teaching status, number of hospital beds, admission type (elective/nonelective) and day (weekend/weekday), bicuspid aortic valve, Elixhauser and Charlson Comorbidity Index scores, and relevant comorbidities (Supplemental Table 4). Abbreviations: aHR, adjusted hazard ratio; SAVR, surgical aortic valve replacement; TAVR, transcatheter aortic valve replacement; uHR, unadjusted hazard ratio.

After propensity score matching 1097 patients (547 TAVR and 550 SAVR), those selected for TAVR had similar 90-day all-cause and heart failure readmissions compared to those selected for SAVR. Readmissions stratified by TAVR versus SAVR are presented in Figure 4.

## Discussion

This analysis of women aged 18-50 years undergoing AVR using the large, nationally-representative NRD yielded several novel findings (Graphical Abstract): (1) the use of TAVR increased whereas the use of SAVR remained similar from 2016 through 2022; (2) compared with SAVR, TAVR was associated with lower rates of in-hospital mortality, AKI, and blood transfusion, and higher rates of heart block and vascular complications; (3) LOS was shorter and nonhome discharges were lower with TAVR compared with SAVR; and (4) 90-day all-cause and heart failure readmissions were similar between TAVR and SAVR.

### Temporal Trends of TAVR versus SAVR

Among women aged 18-50 years, the use of TAVR increased whereas the use of SAVR remained similar. This finding is congruent with a prior retrospective analysis by Alabbadi *et al.,*^30^ who found that among patients ≤60 years old, TAVR use increased significantly from 2013 to 2021, growing at an annual rate of 4.7% (*p* ​< ​0.001). Similarly, a retrospective analysis of the Vizient Clinical Database reported that TAVR use among patients under 65 increased 2.7-fold from 2015 to 2021, nearly equaling SAVR rates (47.5 vs. 52.5%).^31^ The observed increase in TAVR use in our study is likely multifactorial, driven by expanding clinical indications, increased use in patients at higher surgical risk, technological advancements, and growing operator experience, collectively enhancing procedural safety and clinical outcomes.^32^^,^^33^ The minimally invasive nature of TAVR, along with the absence of a long-term anticoagulation requirement—as opposed to mechanical valves—and its reduced procedural burden compared to SAVR, likely made TAVR a more attractive option for younger patients, contributing to its increasing adoption.^33^

### In-Hospital Outcomes

Among women aged 18-50 years, TAVR was associated with lower rates of in-hospital mortality, AKI, and blood transfusion compared to SAVR, but with higher rates of heart block and vascular complications. These results are congruent with a meta-analysis of AVR patients of any age by Siontis *et al.*^34^, who reported a 13% relative risk reduction in all-cause mortality with TAVR compared to SAVR across the spectrum of surgical risk (HR 0.87, 95% CI 0.76–0.99, *p* = 0.038), along with lower rates of AKI and major bleeding, whereas SAVR was associated with fewer vascular complications. Among patients under 60 years old, Gad *et al.*^35^ observed significantly lower rates of AKI (13.0 vs. 21.3%, *p* ​< ​0.001) and blood transfusions (13.2 vs. 31.6%, *p* ​< ​0.001) with TAVR compared to SAVR. The superior in-hospital safety outcomes observed in the TAVR cohort compared to the SAVR cohort likely reflect ongoing advancements in procedural techniques, refinement in patient selection, increased operator expertise, and the widespread adoption of newer-generation transcatheter valve technologies.^34^^,^^35^

Vascular complications are a significant concern in women aged 18-50 years undergoing TAVR, as demonstrated in the present study. These complications may be attributed to the anatomical and physiological factors present in female patients specifically, including smaller caliber vessels and more complex vascular anatomy, which are established predictors of vascular injury.^36^^,^^37^ Although newer devices with smaller sheath sizes have reduced the overall incidence of vascular complications in TAVR, anatomic and procedural challenges persist in this demographic.^36^

The higher incidence of heart block with TAVR compared to SAVR in our study is consistent with findings from prior retrospective studies.^38^ Conduction disturbances following TAVR occur primarily due to the close anatomic proximity of the prosthetic valve to the left bundle branch of the cardiac conduction system.^39^^,^^40^ Device-specific variables, such as valve type and implantation depth, and anatomic variation in the His bundle can predict the risk of conduction disturbances.^39^^,^^40^ Although SAVR-associated conduction disturbances occur due to surgical trauma, TAVR-associated conduction disturbances may result from both device and patient-specific anatomic risks, which may explain the higher rate of conduction disturbances observed in the TAVR cohort.^39^^,^^40^

Although heart block was more common following TAVR, the rate of PPM implantation was similar between TAVR and SAVR. Conduction disturbances are common following TAVR; however, not all cases require PPM implantation. The term “heart block” in our study encompasses a wide spectrum of conduction abnormalities, ranging from first-degree and Mobitz type I second-degree (Wenckebach) atrioventricular block, which are often transient and rarely require PPM implantation, to high-grade atrioventricular block, which more commonly necessitates pacing. Prior studies have shown that persistent left bundle branch block is one of the most common conduction disturbances after TAVR, occurring in 10%–30% of patients, yet only 10%–15% of these ultimately require PPM implantation at 1 ​year.^41^ In addition, younger patients may have a higher rate of recovery from heart block than older patients, thereby reducing the need for a PPM. In a series of children and young adults undergoing TAVR, acute- or late-onset conduction abnormalities developed in 11 patients but resolved in the majority (8/11) during follow-up, with only 1 patient requiring PPM for complete heart block.^42^ These data help explain the apparent discrepancy between the relatively high incidence of heart block events and the lower rates of PPM implantation observed in the TAVR arm of our study. Furthermore, the younger age of our study population may have influenced management decisions, with clinicians favoring close monitoring and a higher threshold for device implantation. This is consistent with prior reports demonstrating notably low PPM implantation rates following TAVR in pediatric populations (3.6%)^42^ and with the lower rates observed in women of childbearing age in the present study compared with those typically reported in older adults.^43^

### LOS and Discharge Disposition

Women aged 18-50 years who underwent TAVR experienced a shorter hospital LOS and were more frequently discharged home compared to those who underwent SAVR. These findings align with previous studies demonstrating that TAVR is associated with reduced hospital LOS and higher rates of routine discharge relative to SAVR among the broader AVR population.^35^^,^^44^ These outcomes are likely attributable to the increased use of conscious sedation and local anesthesia in TAVR procedures, which are associated with fewer procedural complications, reduced need for intensive care, and expedited recovery.^45^ Furthermore, improvements in patient selection and the use of smaller vascular access sheaths may have contributed to earlier discharges in the TAVR group. In contrast, SAVR often necessitates longer recovery periods due to surgical incisions, chest tube placement, and a higher incidence of postoperative complications.^45^ The shorter hospitalization observed with TAVR may also reduce the risk of hospital-acquired infections and physical deconditioning, thereby increasing the likelihood of direct home discharge rather than transfer to a rehabilitation or extended care facility.^45^

### 90-Day Readmission

All-cause and heart failure readmissions within 90 days were similar between TAVR and SAVR in women aged 18-50 years. Previous studies have shown comparable rates of all-cause and heart failure readmissions within 90 days after TAVR versus SAVR in other patient populations, including those aged ≥65 years.^46^^,^^47^ Several potential explanations may account for this finding. First, the patient selection criteria for TAVR and SAVR in this age group may influence outcomes, with careful consideration of comorbidities and severity of AS.^48^ Additionally, advancements in perioperative care and enhanced recovery protocols may mitigate differences between the 2 AVR approaches.^49^ The relatively short follow-up period of 90 days may not have fully captured longer-term differences in readmissions, and further research with extended follow-up is needed to confirm these findings.

### Future Directions

The selection of AVR strategy in women of childbearing age presents unique and complex clinical considerations. Bioprosthetic valves may eliminate the need for lifelong anticoagulation but are subject to structural valve deterioration, often necessitating repeat intervention over time.^50^ If structural valve deterioration occurs after 10-15 years, even with valve-in-valve TAVR performed at that point, these patients may face the risk of outliving 2 bioprosthetic TAVR valves. In contrast, mechanical valves offer superior durability but require indefinite anticoagulation with warfarin, which carries inherent bleeding risks.^50^ In women of reproductive age, warfarin poses additional concerns due to its teratogenic potential during pregnancy.^11^^,^^12^ Furthermore, transitioning to alternative anticoagulation regimens such as low molecular weight heparin during pregnancy is associated with an increased risk of prosthetic valve thrombosis.^11^^,^^12^ These factors underscore the need for individualized, multidisciplinary decision-making in this patient population. Future long-term data on TAVR valve durability will be critical to determining the appropriateness of TAVR in women of childbearing age. In addition, long-term durability data for TAVR-in-SAVR, as well as TAVR-in-TAVR are needed to help determine the lifetime management of aortic valve disease beginning with the index AVR.

New tools are being designed to facilitate repeat transcatheter valve intervention. With future valve-in-valve TAVR expected in younger TAVR patients, the ShortCut catheter (Pi-Cardia, Rehovot, Israel) to facilitate leaflet splitting and prevent coronary obstruction is the subject of an ongoing clinical trial (NCT04952909).

### Limitations and Strengths

This study has several important limitations to acknowledge. First, in a retrospective NRD study using administrative claims codes, incorrect coding could lead to inaccurate data. Second, the retrospective nature of the study makes it subject to inherent selection bias, and RCTs are needed to confirm the present findings. Third, despite propensity score matching resulting in similar measurable baseline characteristics between the 2 study cohorts, there likely remain unmeasured confounders that may affect the findings of this study. Fourth, detailed baseline and procedural characteristics, such as echocardiographic and computed tomographic data, access site, valve type (e.g., balloon-expandable or self-expanding; mechanical or bioprosthetic), valve size, and periprocedural medications, are unavailable in the NRD data set, which can lead to unmeasured bias. Fifth, validated risk scores such as the Society of Thoracic Surgeons score are not captured by the NRD, limiting patient risk assessment and adjustment. Sixth, decisions regarding TAVR versus SAVR may be influenced by patient preferences, institutional expertise, and local protocols, which are not captured in the NRD. Seventh, given the lack of data on out-of-hospital deaths, patients who died at home were counted as patients without a readmission. Finally, our study was limited to in-hospital outcomes and 90-day readmissions. Studies exploring the long-term outcomes of TAVR versus SAVR in women aged 18-50 years, including valve durability, need for reintervention, and late mortality are still needed.

Despite these limitations, this study adds meaningfully to the literature by describing the relative use and comparative outcomes of TAVR versus SAVR in women aged 18-50 years. The NRD is well validated for outcomes studies like this one and undergoes serial data accuracy checks and quality control. In addition, the NRD data are geographically diverse, including a nationally representative sample of centers and operators, and hence reliably reflect real-world practice and outcomes.

## Conclusions

This observational analysis of a large national database demonstrated that TAVR is increasingly performed among women aged 18-50 years and is offered to patients with more comorbidities than those undergoing SAVR. Compared with SAVR, TAVR was associated with lower in-hospital mortality and resource utilization and similar readmissions. These findings are hypothesis-generating, and RCTs exploring the long-term comparative safety and effectiveness of TAVR versus SAVR among women of childbearing age are warranted to confirm these observational findings.

## Ethical Statement

The research reported has adhered to the relevant ethical guidelines.

## Funding

The authors have no funding to report.

## Disclosure Statement

Andrew M. Goldsweig reports consulting for Philips, Abbott, Occlutech, and Conformal Medical and speaking for Philips and Boston Scientific.

Mayra Guerrero has received institutional research grant support from 10.13039/100006520Edwards Lifesciences.

The other authors had no conflicts to declare.
